# Integrated analysis of DNA methylome and transcriptome reveals the differences in biological characteristics of porcine mesenchymal stem cells

**DOI:** 10.1186/s12863-021-01016-8

**Published:** 2021-12-18

**Authors:** Zheng Feng, Yalan Yang, Zhiguo Liu, Weimin Zhao, Lei Huang, Tianwen Wu, Yulian Mu

**Affiliations:** 1 Guangdong Provincial Key Laboratory of Animal Molecular Design and Precise Breeding, Key Laboratory of Animal Molecular Design and Precise Breeding of Guangdong Higher Education Institutes, School of Life Science and Engineering, Foshan University, Foshan, 528231 Guangdong China; 2 Institute of Animal Sciences, Chinese Academy of Agricultural Sciences, Beijing, 100193 China

**Keywords:** DNA methylation, Bone marrow, Umbilical cord, Mesenchymal stem cells, Inbred Wuzhishan miniature pigs

## Abstract

**Background:**

Bone marrow (BM) and umbilical cord (UC) are the main sources of mesenchymal stem cells (MSCs). These two MSCs display significant differences in many biological characteristics, yet the underlying regulation mechanisms of these cells remain largely unknown.

**Results:**

BMMSCs and UCMSCs were isolated from inbred Wuzhishan miniature pigs and the first global DNA methylation and gene expression profiles of porcine MSCs were generated. The osteogenic and adipogenic differentiation ability of porcine BMMSCs is greater than that of UCMSCs. A total of 1979 genes were differentially expressed and 587 genes were differentially methylated at promoter regions in these cells. Integrative analysis revealed that 102 genes displayed differences in both gene expression and promoter methylation. Gene ontology enrichment analysis showed that these genes were associated with cell differentiation, migration, and immunogenicity. Remarkably, skeletal system development-related genes were significantly hypomethylated and upregulated, whereas cell cycle genes were opposite in UCMSCs, implying that these cells have higher cell proliferative activity and lower differentiation potential than BMMSCs.

**Conclusions:**

Our results indicate that DNA methylation plays an important role in regulating the differences in biological characteristics of BMMSCs and UCMSCs. Results of this study provide a molecular theoretical basis for the application of porcine MSCs in human medicine.

**Supplementary Information:**

The online version contains supplementary material available at 10.1186/s12863-021-01016-8.

## Background

Mesenchymal stem cells (MSCs), also known as seed cells, are widely used for tissue repair and regeneration because of their self-renewal and differentiation capacities, together with important immunosuppressive properties and low immunogenicity [1–3]. MSCs were originally isolated from bone marrow (BM). However, the use of BMMSCs is not always acceptable because of the highly invasive donation procedure and significant decline in cell number and proliferative/differentiation capacity with age [4]. In recent years, MSCs have been discovered in almost every tissue of the body, including adult adipose tissue (AT), the placenta, and amniotic fluid [5–7]. Additionally, the umbilical cord (UC) has been introduced as an promising source of MSCs, and UCMSCs have been used in preliminary clinical treatments because they are easily obtained, display less negative effects on the donor than MSCs from other sources, and allow certain ethical questions to be circumvented [8, 9]. Although MSCs derived from different sources share many similar biological characteristics, they also exhibit distinct and unique gene expression and functional properties [10, 11].

The miniature pig (*Sus scrofa*) is an attractive and appropriate large animal model for human diseases because of their anatomical, physiological, and genomic similarities to humans [12, 13]. The inbred Wuzhishan miniature pig has been developed over the last 25 years by the Institute of Animal Sciences, Chinese Academy of Agricultural Sciences. The inbred WZSP line of pigs shows high genetic stability [14], and its inbreeding coefficient reached 0.994 at the 24th generation in 2013 [15]. This line has been widely used to study human diseases, including atherosclerosis, cardiovascular disease, xenotransplantation, and diabetes [16, 17]. Because the quantity of human MSCs that can be obtained is limited, the therapeutic potential of MSCs derived from animal sources other than humans has received wide attention [18–20]. Porcine MSCs are easily obtained, and their morphology and multilineage differentiation potential are similar to those of human MSCs [21]. MSCs derived from inbred WZSPs are highly stable and conducive to establish a reliable system for evaluation of the biological characteristics of porcine MSCs.

DNA methylation is a stable epigenetic modification that regulates many biological processes, including genomic imprinting, X-inactivation, genome stability, and gene regulation [22]. However, there is limited information about regulation of DNA methylation and gene expression in porcine MSCs. In this study, to reveal the molecular mechanism underlying differences in biological characteristics of MSCs, we isolated BMMSCs and UCMSCs from inbred WZSPs. MSCs express mesenchymal markers such as CD29, CD44, CD73, CD90 and CD105 but lack the expression of hematopoetic markers, CD34 and CD45. These markers could be examined by flow cytometry. Genome-wide DNA methylome and transcriptome maps of BMMSCs and UCMSCs were generated by methylated DNA immunoprecipitation sequencing (MeDIP-Seq) and RNA sequencing (RNA-seq), respectively. We identified a set of genes displaying expression and methylation differences between these two MSCs that are critical for regulating the biological functions of these cells. This study provides a molecular theoretical basis for the application of porcine MSCs as a clinical therapy.

## Methods

### Isolation and culture of porcine MSCs

WZSP littermates were purchased from the National Germplasm Resources Center of the Laboratory Miniature Pig, Beijing, China. All animal procedures were approved by the Animal Care and Use Committee of Foshan University and all experiments were performed in accordance with the approved guidelines and regulations. All methods are reported in accordance with ARRIVE guidelines (https://arriveguidelines.org) for the reporting of animal experiments. The pigs were injected intravenously with propofol (2 mg/kg) to induce full anesthesia. UCMSCs were isolated from the umbilical cords of four WZSP littermates on the day of birth, and BMMSCs were isolated from the bone marrow of the same individuals at 42 days after birth. To isolate UCMSCs, umbilical cords were cut into 1–2 mm^2^ pieces, attached, and cultured. To isolate BMMSCs, bone marrow was extracted and centrifuged for 5 min at 4 °C with 1000 rpm. The isolated MSCs were cultured in DMEM/F12 medium (Gibco) with 20% fetal bovine serum (Gibco), 50 units/mL penicillin G, and 50 μg/mL streptomycin, and incubated at 37 °C in 5% CO_2_ in a humidified incubator. The medium was replaced every 3 days.

### FCM analysis of cell surface antigen expression

FCM was used to analyze the surface marker phenotypes of MSCs, as described in our previous reports [23]. Cells were harvested by exposure to 0.05% trypsin-EDTA for 3 min at 37 °C, followed by washing and fixation. MSCs were resuspended in 1% (w/v) bovine serum albumin (Sigma) for 30 min at room temperature to block non-specific binding sites. After blocking, the BMMSCs were incubated with CD29 (VMRD), CD44 (VMRD), CD45 (VMRD), and FITC-anti-human CD34/PE-anti human CD90 (eBioscience) monoclonal antibodies at room temperature for 20 min. The UCMSCs were incubated with CD31, CD45 (Veterinary Medical Research & Development, VMRD), and FITC-anti-human CD34/PE-anti human CD90 (eBioscience) monoclonal antibodies at room temperature for 20 min. The CD29, CD44, and CD45 groups were then stained with rat anti-mouse IgG1-FITC (IVGN), goat anti-mouse IgG2a-PE secondary antibody (IVGN), and anti-mouse IgM-PE (eBioscience), respectively, at room temperature for 20 min. FCM data acquisition and analysis were performed with a BD FACS Calibur Flow Cytometer and Cell Quest software. For the negative control, cells were incubated only with Dulbecco’s phosphate-buffered saline. Each FCM experiment was performed in triplicate.

### Adipogenic and osteogenic differentiation of porcine BMMSCs and UCMSCs

The differentiation of porcine BMMSCs and UCMSCs was performed as described previously [24]. Briefly, to evaluate the differentiation ability of MSCs in vitro, we replaced the DMEM/F12 medium with an adipogenic/osteogenic differentiating medium (Gibco) when cells reached 80% confluency. The cells were cultured at 37 °C in 5% (vol/vol) CO2 in 100% humidified air. Cells were cultured for 2 to 3 weeks before collection, with the medium changed every 3 days. At 2 or 3 weeks, Oil red O was used to assess adipogenic differentiation, and Alizarin Red S staining was used to evaluate osteogenic differentiation. Adipogenic and osteogenic differentiation assays were performed three times.

### MeDIP-seq

Genomic DNA was isolated using an E.Z.N.A. HP Tissue DNA Midi Kit (Omega) and sonicated to 100–500-bp fragments with a Bioruptor Sonicator (Diagenode). Four BMMSC and four UCMSC DNA samples were pooled by homogeneous mixing prior to MeDIP-seq. The libraries were constructed following the manufacturer’s instructions, as described in our previous reports [25, 26], and sequenced on an Illumina HiSeq 2000 with 49-bp paired-end reads.

### MeDIP-seq data analysis

After filtering out low-quality reads that contained more than 5 ‘N’s or had low quality values (Phred score < 5) for over 50% of the sequence, clean reads were aligned to the pig reference genome (*Sus scrofa* Sscrofa11.1) downloaded from the USCS database, allowing up to two mismatches, in SOAP2 (v2.21) [27]. Reads mapping to the same genomic location were regarded as possible clonal duplicates resulting from PCR amplification biases. To avoid stochastic sampling drift, we filtered out CpG sites with a coverage depth of less than 10 reads [28]. Annotation information for CpG Islands (CpGi) in the pig genome was downloaded from the UCSC public FTP site. Model-based analysis of ChIP-Seq (MACS v1.4.2) (http://liulab.dfci.harvard.edu/MACS/) was used to scan for methylation peaks in the pig genome with default parameters (−EXTSIZE 200; –QVALUE 0.01) [29]. The methylation level at each peak was calculated using the RPKM method. DMRs were identified with the criteria of FDR adjusted *P* < 0.05 by edgeR (exact test for negative binomial distribution) integrated in MeDIPs.. We defined regions 2 kb upstream of the TSS as promoters and regions from the TSS to the TTS as the gene body. Promoters that contained one or more DMRs were considered differentially methylated promoters for further analysis.

### Transcriptome sequencing and data analysis

RNA from BMMSCs and UCMSCs was isolated using Trizol reagent (Invitrogen, Carlsbad, CA, USA), treated with DNase I (Qiagen, Basel, Switzerland), and then cleaned using an RNAeasy MiniElute Cleanup kit (Qiagen, Basel, Switzerland). The integrity of total RNA was checked with an Agilent 2100 Bioanalyze instrument (Agilent Technologies, Palo Alto, CA, USA), and only RNA samples with a RNA integrity number score > 8 were subjected to sequencing. Equal amounts of RNA from four BMMSC and UCMSC samples were pooled. Beads with oligo (dT) were then used to isolate poly (A) mRNA after total RNA was collected. Fragmentation buffer was added to break up the mRNA. Using these short fragments as templates and random hexamer primers, first-strand cDNA was synthesized. Second-strand cDNA was synthesized using buffer, dNTPs, RNaseH, and DNA polymerase I. Short fragments were purified using a QiaQuick PCR extraction kit and resolved with EB buffer for end repair and poly (A) addition. The short fragments were then connected with sequencing adaptors. For PCR amplification, we selected suitable fragments to serve as templates, with respect to the result of agarose gel electrophoresis. The libraries were sequenced using an Illumina HiSeq 2000 to generate 90-bp paired-end reads.

After trimming adaptor sequences and removing low-quality reads, clean reads were mapped to a *Sus scrofa* reference genome using SOAP2 (v2.21) and allowing up to three mismatches [27]. RPKM values were used to represent the expression level of each gene. Genes differentially expressed between BMMSCs and UCMSCs were identified using the exact test for negative binomial distributions. Genes with FDRs < 0.05 and |log2 FC| ≥ 1 were considered differentially expressed.

### GO enrichment analysis

Functional enrichment analysis was performed using the DAVID (Database for Annotation, Visualization, and Integrated Discovery) web server (http://david.abcc.ncifcrf.gov/) [30]. Genes with differentially methylated promoters were mapped to their human orthologs and submitted to DAVID for GO enrichment analysis.

### RT-qPCR

RT-qPCR was performed using three biological replicates for each MSCs and three technical replicates per biological sample. Total RNA was extracted using an RNA Extraction Kit (BioTeke). First-strand cDNA was synthesized using oligo (dT)18 primers provided in the RevertAid First Strand cDNA synthesis kit (Thermo). qPCR was performed on an ABI 7500 machine using a SYBR Premix Ex Taq kit (TaKaRa), and the glyceraldehyde-3-phosphate dehydrogenase gene (*GAPDH*) was used as endogenous control gene. Relative expression levels of mRNAs were calculated using the 2^-ΔΔCt^ method. Primer sequences are shown in Additional file: Table S4.

### Sequenom MassARRAY quantitative methylation analysis

DNA isolated from UCMSCs and BMMSCs was treated with sodium bisulfite using an EZ DNA Methylation-Gold Kit (ZYMO Research) according to the manufacturer’s instructions. A quantitative analysis of DMRs was performed using the Sequenom MassARRAY platform (CapitalBio, Beijing, China) [31]. Specific primers were designed using EpiDesigner software (Sequenom), and the quantitative results for each CpG or multiple CpGs were analyzed in EpiTyper v1.0 (Sequenom). Primer sequences are shown in Additional file: Table S4.

### Statistical analysis

A two-tailed Student t- test or One-way ANOVA followed by Tukey test was used to compare significant differences between groups. A *P* value of *P* < 0.05 was considered statistically significant.

## Results

### Isolation and identification of porcine BMMSCs and UCMSCs

**Fig. 1:**
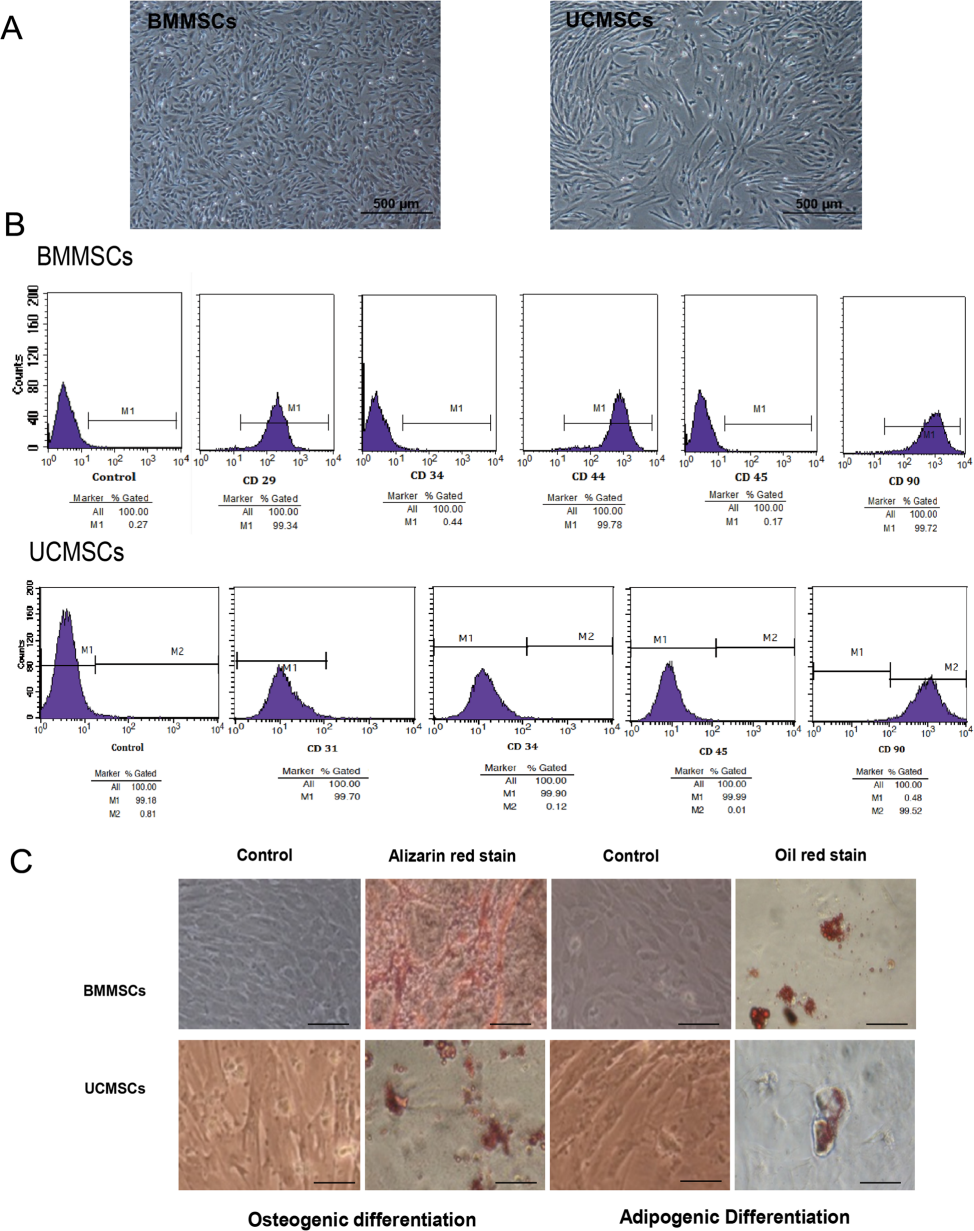
Isolation and identification of porcine BMMSCs and UCMSCs. **A** The fibroblast-like morphology of porcine MSCs. **B** FCM analysis of surface markers expressed on MSCs. Fluorescence in the range of M1 was considered an indicator that cells were recognized by the directed antibody. Autofluorescence intensity was less than 10^1^; cells will fluorescence below this threshold were considered negative. **C** Osteogenic and adipogenic differentiation potential of porcine BMMSCs and UCMSCs. Calcium deposits in osteocytes and lipid droplets in adipocytes were stained red with Alizarin Red and Oil Red O, respectively. Scale bars, 50 μm

We isolated BMMSCs and UCMSCs from inbred WZSPs. Adhesion of BMMSCs to plastic flasks was observed 24 h after isolation. As the culture continued, adherent cells displayed a scattered distribution, growing in isolated clones. UCMSCs gradually grew outward from the UC tissues after 7 days. The morphology of UCMSCs was similar to that of BMMSCs: the majority of the cells were fusiform and their nucleoli were clear. The passaged cells reached 90% confluency after approximately 3 days (Fig. 1A).

Flow cytometry (FCM) analysis was performed to confirm the surface marker characteristics of MSCs. In BMMSCs and UCMSCs, stem cell surface markers CD29, CD44, and CD90 were detected, whereas leucocyte marker CD45 and hematopoietic lineage marker CD34 were not (Fig. 1B). The UCMSCs were positive for CD90, but negative for CD34, CD45, and endothelial marker CD31 (Fig. 1B). The in vitro potential of BMMSCs and UCMSCs to differentiate into osteogenic and adipogenic lineages was also evaluated. We observed an increase in the number of calcified nodules on the surfaces of MSCs with induction of osteoblast differentiation. On the 21st day after induction of osteogenic differentiation, the morphology of MSCs significantly changed to include the substantial accumulation of orange sediment (Fig. 1C). The calcified nodules on BMMSCs were much more obvious than those on UCMSCs. On the 21st day after induction of adipogenic differentiation, numerous intracellular lipid droplets formed (Fig. 1C), and the lipid droplets in BMMSCs were much more obvious than those in UCMSCs. These results indicated that both MSCs had the potential for osteogenic and adipogenic differentiation, but that the differentiation ability of BMMSCs was stronger than that of UCMSCs.

### DNA methylome and transcriptome profiles for porcine BMMSCs and UCMSCs

We carried out MeDIP-seq and RNA-seq analyses to develop genome-wide DNA methylome and transcriptome profiles for porcine BMMSCs and UCMSCs. Approximately 7.2 Gb clean reads were generated for each MeDIP-seq library. Of all reads from the BMMSCs and UCMSCs, 75.52 and 76.42%, respectively, could map to the pig reference genome. For each RNA-seq library, approximately 4.8 Gb of clean reads were obtained. Clean reads from the BMMSCs and UCMSCs aligned to 59.90 and 59.83%, respectively, of the pig reference genome. After removing duplicate reads, the remaining uniquely aligned reads were used for further analyses.

### Methylome characteristics of porcine BMMSCs and UCMSCs

**Fig. 2:**
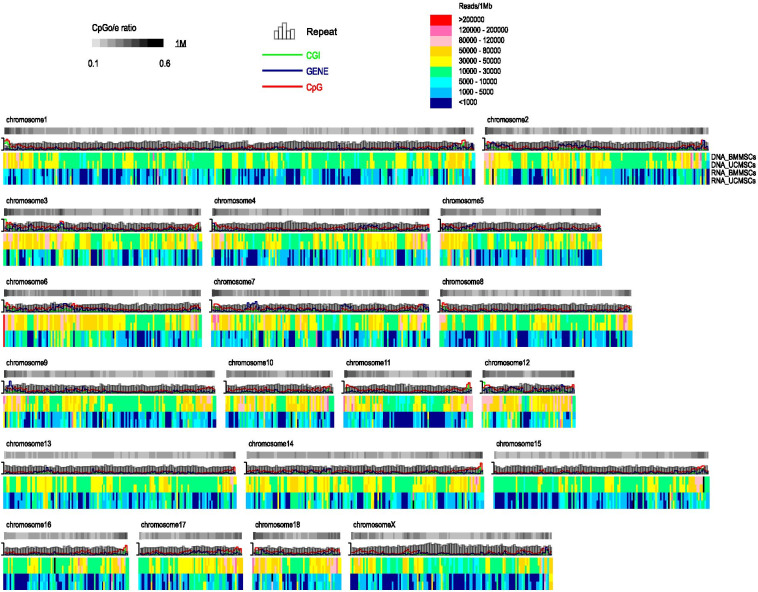
DNA methylome and transcriptome maps of porcine MSCs. The distribution of DNA methylation and levels of gene expression throughout the pig chromosomes were determined. To compare DNA methylation and transcription levels in BMMSCs and UCMSCs, read depths were normalized to the average number of reads in each sample. A 1-Mb sliding window was used to smooth the distribution. Repeat elements, CGI length, gene density, CpG number, and CpGo/e ratio were all calculated in the 1-Mb sliding window

**Fig. 3:**
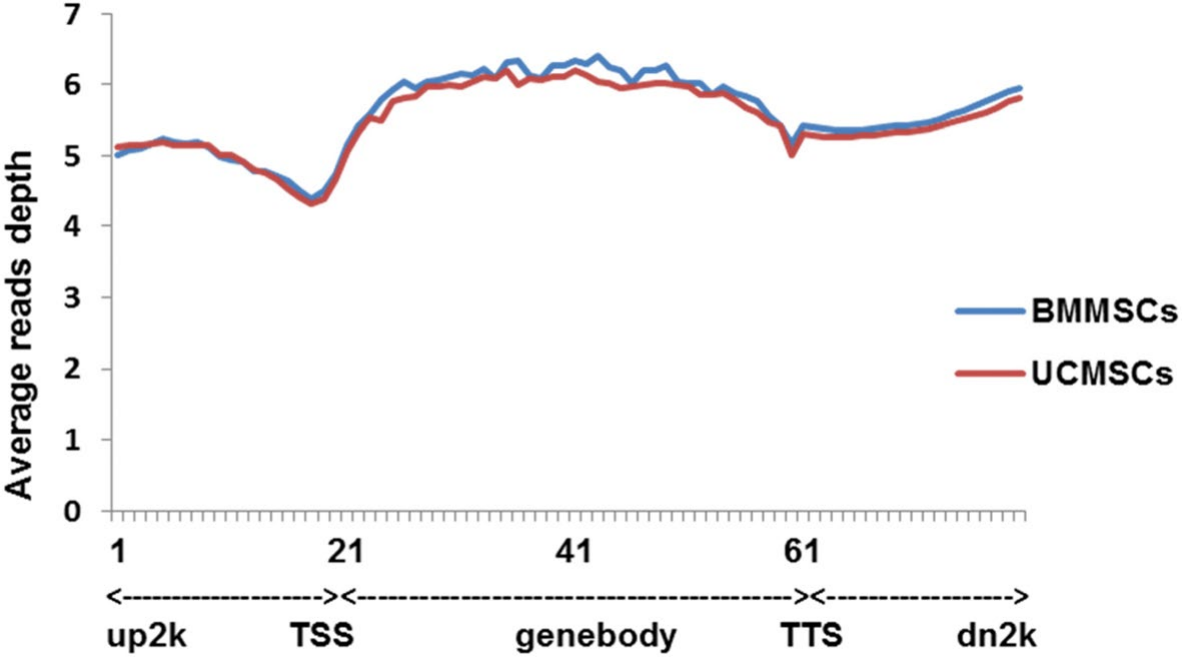
DNA methylation distribution around gene bodies and flanking regions in porcine MSCs. The 2-kb regions upstream and downstream of TSSs and TTSs, respectively, were split into 20 non-overlapping windows, and the body of each gene was split into 40 equal windows. Average alignment depth was calculated for each window. The Y-axis is the average read depth for each window

We first analyzed the genome-wide DNA methylation patterns of porcine MSCs (Fig. 2) and found that methylation level negatively correlated with repeat length (Pearson’s *r* = − 0.248, ***P*** < 0.001) and positively correlated with gene number (Pearson’s *r* = 0.335, ***P*** < 0.001), CpG island (CGI) length (Pearson’s *r* = 0.482, ***P*** < 0.001), CpG site number (Pearson’s *r* = 0.777, ***P*** < 0.001), and especially with observed over expected CpG ratio (CpGo/e) (Pearson’s *r* = 0.790, ***P*** < 0.001). We further analyzed methylation of the 2-kb regions upstream of the transcription start sites (TSSs), the gene body, and 2-kb regions downstream of the transcription termination sites (TTSs) in MSCs (Fig. 3). The TSSs in both MSCs displayed low methylation, whereas the DNA methylation levels in gene bodies were relatively constant and much higher than those in the 5′ and 3′ flanking regions. These results were consistent with previous reports [25].

### Promoter methylation and transcriptional repression in MSCs

**Fig. 4:**
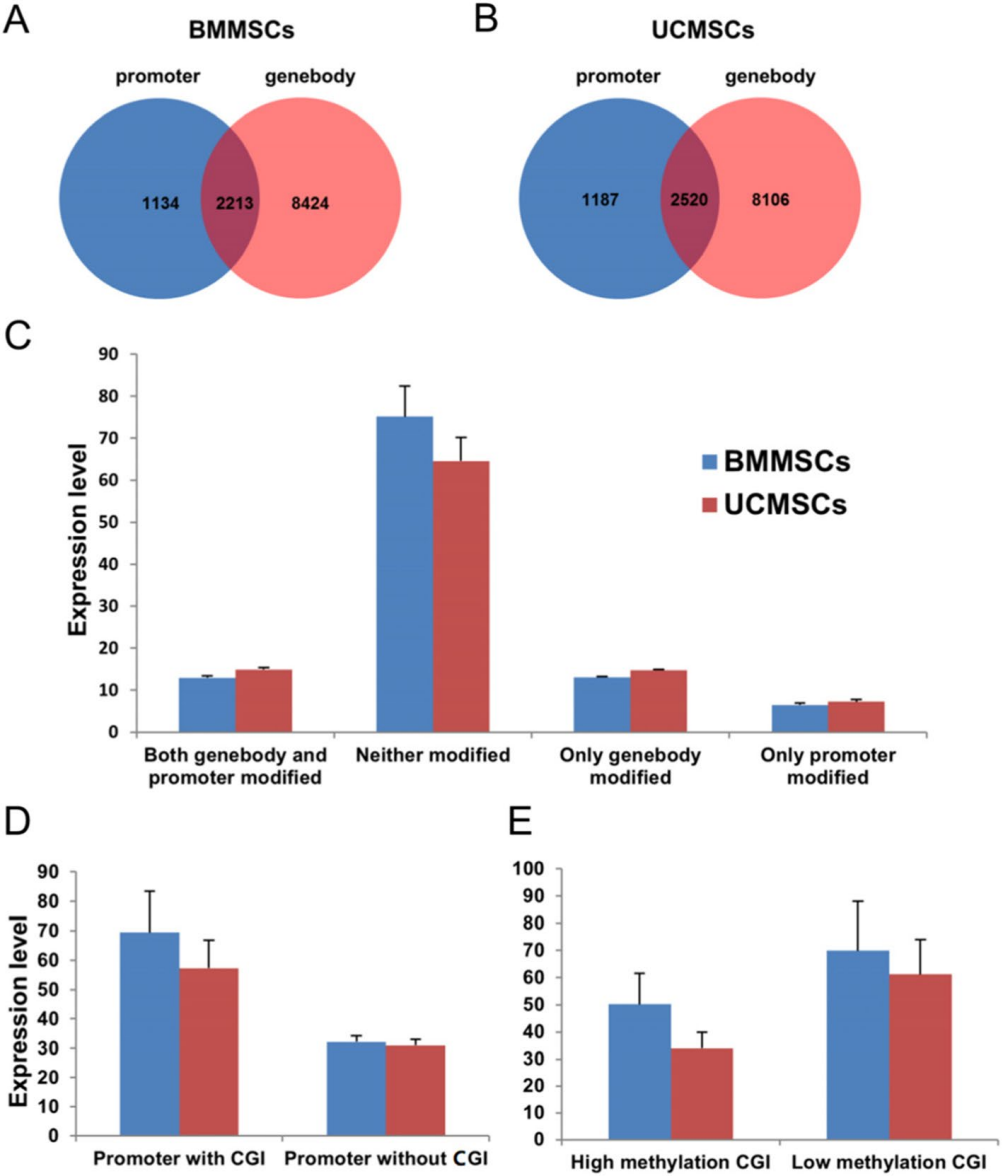
Promoter methylation and transcriptional repression in porcine MSCs. **A** The number of gene promoters and/or gene bodies showing methylation modifications in BMMSCs. **B** The number of gene promoters and/or gene bodies showing methylation modifications in BMMSCs. **C** Comparison of expression between genes showing promoter and/or gene body methylation. **D** Comparison of expression between genes with promoter CGIs and genes without promoter CGIs. (E) Comparison of expression between genes with different methylation levels at promoter CGIs

Methylation peaks were detected across different genomic elements. Reads per kilobases per million reads (RPKM) values were used to evaluate the methylation level at each peak. A total of 150,690 and 161,105 methylation peaks were generated, with average lengths of 1462 and 1466 bp in BMMSCs and UCMSCs, respectively, covering 9.74 and 10.44%, respectively, of the *Sus scrofa* genome. We classified genes into four groups according their methyl modifications: (I) only the promoter was modified; (II) only the gene body was modified; (III) both were modified; and (IV) neither promoter nor gene body were modified. The numbers of genes classified into these four methylation types in BMMSCs were 1134, 8424, 2213, and 8656, respectively (Fig. 4A), and the numbers in UCMSCs were 1187, 8106, 2520, and 8614, respectively (Fig. 4B). The expression levels of genes in group IV were significantly higher than those of genes in the other three groups, whereas the genes in group I exhibited the lowest expression levels (Fig. 4C). These results implied that both promoter and gene body methylation patterns could affect gene expression. We analyzed the effects of promoter CGIs on gene expression and found that the expression levels of genes without promoter CGIs were significantly lower than those of genes with promoter CGIs (Fig. 4D). Meanwhile, we found genes with low levels of methyl modifications at promoter CGIs showed significantly higher expression levels than genes with high levels of methyl modifications at promoter CGIs (Fig. 4E), suggesting that methylation of CGIs also regulated gene expression in MSCs.

### Differentially expressed genes (DEGs) in BMMSCs and UCMSCs

**Fig. 5:**
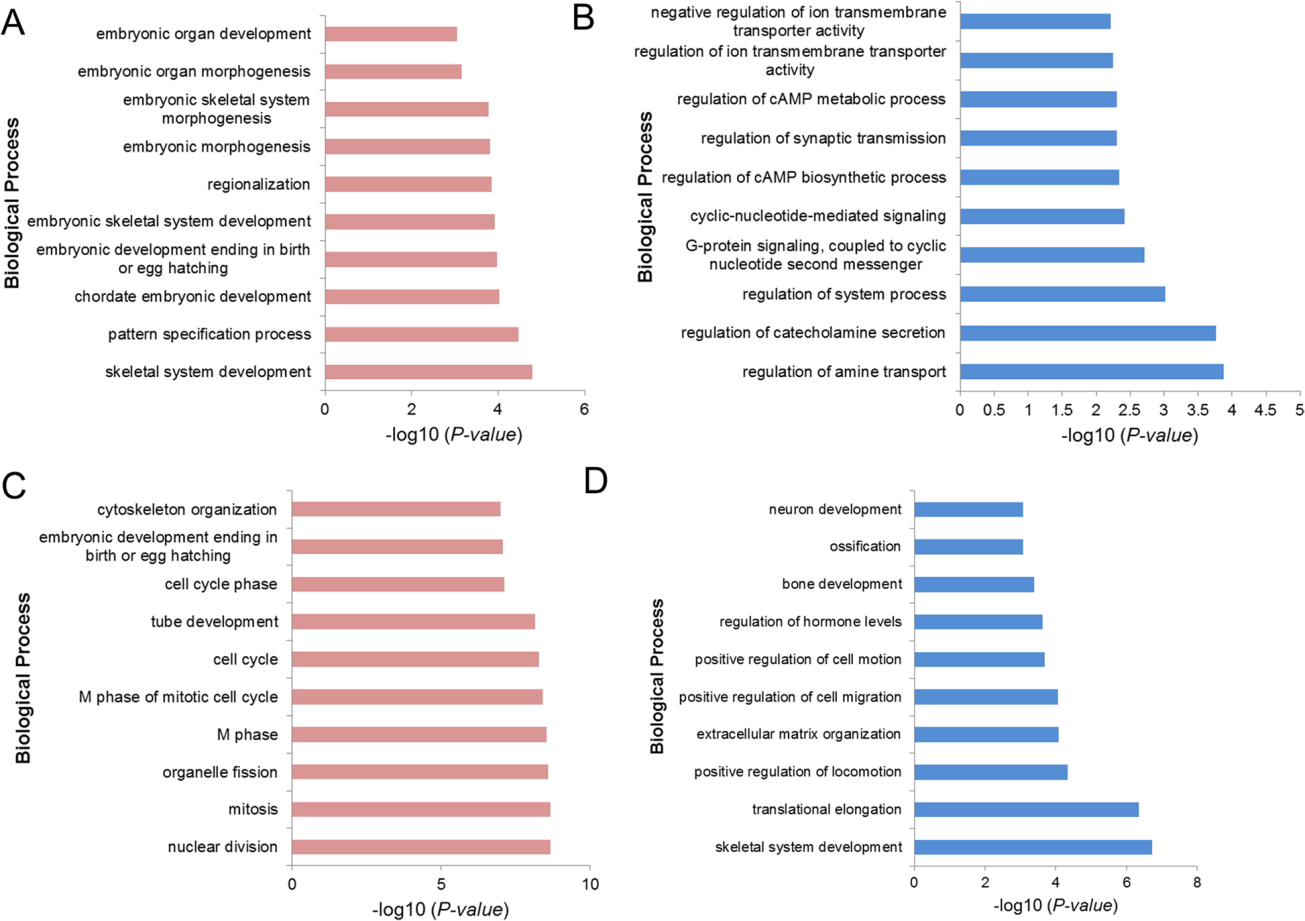
GO functional enrichment analysis of DEGs in BMMSCs and UCMSCs. **A**–**B** The top 10 biological process terms significantly enriched for hypermethylated (**A**) and hypomethylated (**B**) genes in UCMSCs compared to those in BMMSCs. **C**, **D** The top 10 biological process terms significantly enriched for upregulated (**C**) and downregulated (**D**) genes in UCMSCs compared to those in BMMSCs

We next compared differences in DNA methylation and gene expression between porcine BMMSCs and UCMSCs. A total of 587 genes showed differential methylation at promoter regions; 280 of these genes were hypermethylated and 307 were hypomethylated in UCMSCs (Additional file: Table S1). Gene Ontology (GO) enrichment analysis revealed that the hypermethylated genes were significantly associated with skeletal system development, pattern specification processes, and chordate embryonic development (Fig. 5A). In contrast, hypomethylated genes were significantly enriched in regulation of amine transport, catecholamine secretion, and system processes, as well as G-protein signaling coupled to cyclic nucleotide second messengers (Fig. 5B).

We also identified 1979 DEGs in BMMSCs and UCMSCs (Additional file: Table S2). Compared with BMMSCs, 1407 genes were upregulated and 572 genes were downregulated in UCMSCs. GO enrichment analysis revealed that the upregulated genes were significantly enriched in functions related to nuclear division, mitosis, organelle fission, and cell cycling (Fig. 5C), implying that UCMSCs have higher cell proliferative capacity than BMMSCs. The downregulated genes were significantly enriched in functions related to skeletal system development, translational elongation cell migration, cell adhesion, ossification, and metabolism-related processes (Fig. 5D). These DEGs suggested characteristics of MSCs that were dependent on cellular source.

We found 102 genes that had both expression and promoter methylation differences. Thirty-six of these genes were hypermethylated and downregulated in BMMSCs, including C8ORF73, AOC3, FGF21, AC005841.1, CLDN4, TRPV2, MUC20, SERPINB5, CACNA1G, KCNH2, MCAM, BVES, ULBP3, CSMD2, PCDHGA7, TMEM200B, HTR1B, SLC22A18, CTF1, GPR44, CLSTN3, GPSM3, SPRY4, HOXD11, HOXC5, KIAA0895, CNTFR, ZBTB39, PEMT, FOXL1, FUT1, PMEPA1, RCSD1, DAB2IP, TNFRSF10B, and AC024575.1. In contrast, 15 of these genes were hypermethylated and downregulated in UCMSCs, including GATM, ADAMTS16, LPAR1, ITIH5, CFI, PTN, MLANA, FCRL1, CWH43, PAM, MOXD1, C6orf204, ARNTL2, SYN1, and SLC9A9.

### Validation of the MeDIP-seq and RNA-seq data

**Fig. 6:**
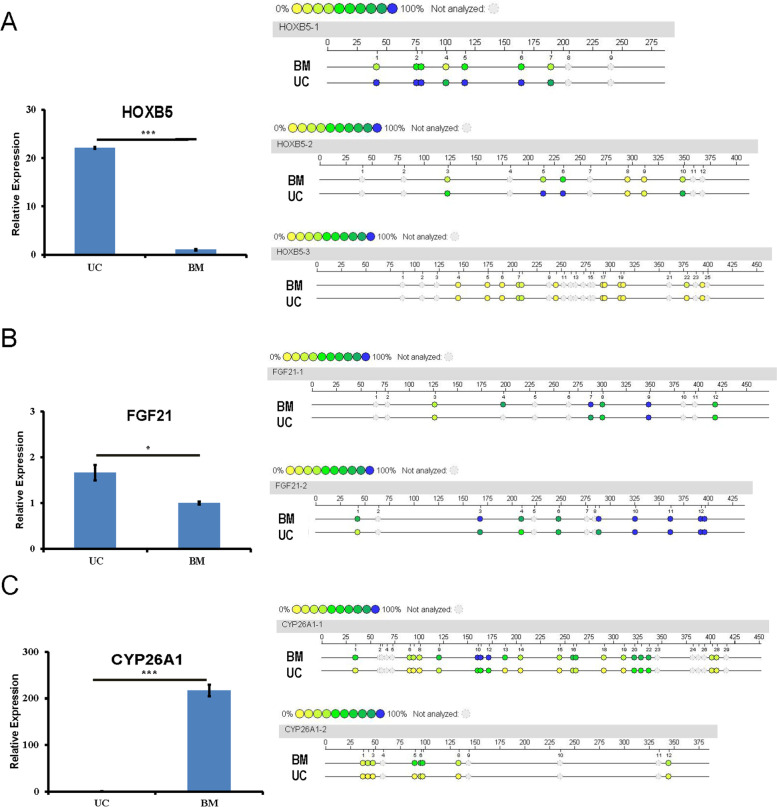
RNA-seq and MeDIP-seq data validation by RT-qPCR and Sequenom MassARRAY, respectively. The expression and promoter methylation levels of three representative genes (HOXB5, FGF21, and CYP26A1) were validated by RT-qPCR and Sequenom MassARRAY, respectively. **A** HOXB5, **B** FGF21, and **C** CYP26A1. The expression levels of these three genes in BMMSCs and UCMSCs are shown in the left panel. Error bars denote standard errors of means (* represents *P* < 0.05, *** represents *P* < 0.001). The right panel shows the Sequenom MassARRAY results. Each dot corresponds to one CpG position in the genome sequence. The colored bar summarizes the methylation level at that position, with blue indicating methylation (100%) and yellow indicating a lack of methylation (0%). Both analyses were performed with three biological replicates for each MSC. Results of the validation of other DEGs or differentially methylated promoter regions are shown in Additional file: Table S3

The degree of methylation in 31 differentially methylated regions (DMRs) in the promoters of 15 genes was verified by Sequenom MassARRAY methylation analysis (Fig. 6 and Additional file: Table S3), and the expression levels of 3 DEGs were validated by real-time quantitative PCR (RT-qPCR, Fig. 6). These results agreed with those of the MeDIP-seq and RNA-seq analyses, establishing the reliability of our omic data.

## Discussion

The biological characteristics of MSCs derived from different sources can differ in proliferation, differentiation, and migration abilities that affect their tissue repair capacity [1–3]. Porcine MSCs are easily obtained, and their morphology and differentiation potential are similar to those of human MSCs. The inbred WZSP line is an ideal large animal model with high genetic stability [14], providing an excellent model to understand the molecular characteristics of MSCs. To explore the biological characteristics and regulatory mechanisms of MSCs derived from different sources, we isolated BMMSCs and UCMSCs from WZSPs and created genome-wide DNA methylome and transcriptome maps of these two MSCs.

Our results showed that porcine MSCs had DNA methylation patterns similar to those in cells from other pig tissues [25, 26, 28]: TSSs maintained a low methylation status, and gene bodies exhibited a much higher level of DNA methylation than the 5′ and 3′ flanking regions. Genome-wide integrated DNA methylome and transcriptome maps of porcine MSCs showed that gene expression was affected by both promoter and gene body methylation, and confirmed that promoter methylation represses gene expression [32, 33]. Most CpGs in mammalian genomes are methylated, whereas CpGs in CGIs are usually unmethylated. However, methylated CGIs are associated with some normal biological processes such as X chromosome inactivation and gene imprinting [34]. In this study, we found that the expression levels of genes without promoter CGIs were significantly lower than those of genes with promoter CGIs. Additionally, promoter CGI methylation levels showed a negative correlation with gene expression levels. These results indicated that CGI methylation might regulate gene expression in MSCs. However, this regulatory mechanism is yet to be defined.

MSCs derived from different sources can also manifest unique molecular characteristics. We identified 587 genes displaying promoter methylation differences and 1979 genes displaying expression differences between BMMSCs and UCMSCs. In total, 102 genes showed both expression and promoter methylation differences. Enrichment analysis revealed that DEGs were functionally related to the biological characteristics of MSCs. Skeletal system development was the most significantly associated biological process for both hypermethylated genes (e.g., Homeobox genes) and downregulated genes (e.g., pleiotrophin [PTN], RBP4) in UCMSCs. Homeobox genes are master developmental control genes that act at the top of genetic hierarchies to regulate aspects of morphogenesis and cell differentiation in animals [35]. PTN showed a higher expression level and lower degree of promoter methylation in BMMSCs than in UCMSCs. This gene plays an important role in bone formation by mediating the recruitment and attachment of osteoblasts/osteoblast precursors to appropriate substrates for the deposition of new bone [36]. These results indicated that BMMSCs have much higher osteogenic differentiation potential than UCMSCs. A previous study also showed that the osteoblast differentiation of UCMSCs was less efficient, even after the addition of 1.25-dihydroxyvitamin D3, a potent osteoinductive substance [37].

Compared with UCMSCs, the inter-alpha (globulin) inhibitor H5 (ITIH5) gene showed a higher level of expression and lower degree of promoter methylation in BMMSCs. *ITIH5* was highly expressed in human adipocytes and adipose tissue, and its expression was higher in obese subjects and was reduced with diet-induced weight loss [38]. Fibroblast growth factor 21 (FGF21), an endocrine regulator of lipid metabolism, caused a dramatic decline in fasting plasma glucose, fructosamine, triglycerides, insulin, and glucagon levels when administered daily for 6 weeks to diabetic rhesus monkeys [39, 40]. Compared with BMMSCs, *ITIH5* and *FGF21* showed higher gene expression and lower promoter methylation levels in UCMSCs. These results indicated that BMMSCs have greater adipogenic differentiation capacity than UCMSCs.

We observed that cell cycle-related genes such as CTF1, DAB2IP, and CACNA1G were significantly upregulated and hypomethylated in UCMSCs. Cardiotrophin 1 (CTF1) stimulates the proliferation of cardiomyocytes [41] and plays an important role in cardiac repair in infarcted hearts [42]. DAB2 interacting protein (DAB2IP) is a newly described member of the Ras GTPase-activating protein family and plays an important role in maintaining cell homeostasis and regulating cell proliferation, survival, and death [43]. Calcium channel, voltage-dependent, T type, alpha 1G subunit (CACNA1G) is a T-type calcium channel gene. Hypermethylation of CACNA1G has been shown in various human tumors and may cell proliferation and apoptosis [44]. Results from our study indicate that UCMSCs have higher cell proliferative capacity than BMMSCs.

The extent of tight junction formation is one of many factors that regulate motility, invasion, and metastasis. A member of the claudin family of proteins, claudin 4 (CLDN4) is required for the formation and maintenance of tight junctions [45]. The forkhead box L1 (FOXL1) protein belongs to the forkhead box (Fox) family of transcription factors. Its overexpression inhibits tumor cell growth, migration, and invasion of renal and pancreatic cancer cells [46, 47]. Compared with *CLDN4* and *FOXL1* in BMMSCs, both genes displayed higher levels of expression and lower levels of promoter methylation in UCMSCs, suggesting a difference in the migration potential of these porcine MSCs.

G protein-coupled receptor 44 (GPR44) plays a major role in the activation and chemotaxis of Th2 cells, eosinophils, and basophils [48], whereas G-protein signaling modulator-3 (GPSM3) is known to bind Gαi·GDP subunits and free Gβ subunits during Gγ dimer formation. GPSM3 is an important regulator of monocyte function, including their differentiation, chemotaxis, and survival in vitro and in vivo; deficiency in this protein is protective against acute inflammatory arthritis [49]. UL16 binding protein 3 (ULBP3), an MHC class I-related molecule, can bind human cytomegalovirus glycoprotein UL16 and activate natural killer cells [50]. Lower expression and higher methylation of *GPR44*, *GPSM3*, and *ULBP3* in UCMSCs compared with BMMSCs suggested that the two MSCs have different immunogenic potential.

## Conclusions

In summary, we generated the first global integrated DNA methylation and transcription maps of porcine MSCs, illuminating the critical role of DNA methylation in determining differences in the biological characteristics of BMMSCs and UCMSCs. This study provides a molecular theoretical basis for the application of human MSCs. However, the functions of genes responsible for differences in BMMSCs and UCMSCs still need to be deciphered at multiple levels.

## Supplementary Information

**Additional file 1. **The datasets used and/or analysed during the current study are available in the article. **Table S1**. List of genes displaying differences in promoter methylation between BMMSCs and UCMSCs. **Table S2**. List of DEGs in BMMSCs and UCMSCs. **Table S3**. Validation of DMPs by Sequenom MassARRAY. **Table S4**. Primer sequences for the RT-qPCR and Sequenom MassARRAY analyses. All animal procedures were approved by the Animal Care and Use Committee of Foshan University and all experiments were performed in accordance with the approved guidelines and regulations.

## Data Availability

The datasets generated during the current study are available in the figshare repository, https://figshare.com/articles/dataset/Mscs_sequencing_data/16862959 or https://figshare.com/search?q=10.6084%2Fm9.figshare.16862959 and could also be found with searching the DOI (10.6084/m9.figshare.16862959) in the searchign box in the first page of the website https://figshare.com.

## Abbreviations

BM: Bone marrow
UC: Umbilical cord
MSCs: Mesenchymal stem cells
DAVID: Database for Annotation, Visualization, and Integrated Discovery
WZSP: Wuzhishan pig
CGI: Cytosine phosphate guanine island
CPG: Cytosine phosphate guanine
FDR: False discovery rate
GO: Gene Ontology
RT-qPCR: Real-time quantitative PCR
DMRs: Differentially methylated regions
RPKM: Reads per kilobase per million reads
TSS: Transcription start site
TTS: Transcription termination site

## References

[1] Kolf CM, Cho E, Tuan RS (2007). Mesenchymal stromal cells - biology of adult mesenchymal stem cells: regulation of niche, self-renewal and differentiation. Arthritis Res Ther.

[2] Li N, Hua JL (2017). Interactions between mesenchymal stem cells and the immune system. Cell Mol Life Sci.

[3] Shi YF, Hu GZ, Su JJ, Li WZ, Chen Q, Shou PS, Xu CL, Chen XD, Huang Y, Zhu ZX (2010). Mesenchymal stem cells: a new strategy for immunosuppression and tissue repair. Cell Res.

[4] Romanov YA, Svintsitskaya VA, Smirnov VN (2003). Searching for alternative sources of postnatal human mesenchymal stem cells: candidate MSC-like cells from umbilical cord. Stem Cells.

[5] Semenov OV, Sonja K, Mariluce R, Nikolas Z, Roland Z, Zisch AH, Antoine M (2010). Multipotent mesenchymal stem cells from human placenta: critical parameters for isolation and maintenance of stemness after isolation. Am J Obstet Gynecol.

[6] Ivana A, Liborio S, Yuji K, Seongjin Y, Naoki T, Bae EC, Chheda SH, Weinbren NL, Borlongan CV (2011). Amniotic fluid as a rich source of mesenchymal stromal cells for transplantation therapy. Cell Transplant.

[7] Urrutia DN, Caviedes P, Mardones R, Minguell JPJ, Vega-Letter AM, Jofre CM (2019). Comparative study of the neural differentiation capacity of mesenchymal stromal cells from different tissue sources: an approach for their use in neural regeneration therapies. PLoS One.

[8] Chandravanshi B, Bhonde RR (2018). Human umbilical cord-derived stem cells: isolation, characterization, differentiation, and application in treating diabetes. Crit Rev Biomed Eng.

[9] Lindenmair A, Hatlapatka T, Kollwig G, Hennerbichler S, Gabriel C, Wolbank S, Redl H, Kasper C (2012). Mesenchymal stem or stromal cells from amnion and umbilical cord tissue and their potential for clinical applications. Cells.

[10] Si YL, Zhao YL, Hao HJ, Fu XB, Han WD (2011). MSCs: biological characteristics, clinical applications and their outstanding concerns. Ageing Res Rev.

[11] Cho KA, Park M, Kim YH, Woo SY, Ryu KH (2017). RNA sequencing reveals a transcriptomic portrait of human mesenchymal stem cells from bone marrow, adipose tissue, and palatine tonsils. Sci Rep.

[12] Vodička P, SMETANA K, Dvořánková B, Emerick T, Yingzhi Z, Ourednik J, Ourednik V, Motlík J, XU (2010). The miniature pig as an animal model in biomedical research. Ann N Y Acad Sci.

[13] Wang S, Liu Y, Fang D, Shi S (2010). The miniature pig: a useful large animal model for dental and orofacial research. Oral Dis.

[14] Fang XD, Mu YL, Huang ZY, Li Y, Han LJ, Zhang YF, et al. The sequence and analysis of a Chinese pig genome. Gigascience. 2012;1(1):16. 10.1186/2047-217X-1-16 PMC3626506 23587058

[15] Mu Y-l, Liu L, Feng S-t, Wu T-w, Li K, Li J-y, He W, Gao Q, Zhou W-f, Wei J-L (2015). Identification of the miniature pig inbred line by skin allograft. J Integr Agric.

[16] Zhao YQ, Xiang L, Liu YQ, Niu MM, Yuan JF, Chen H (2018). Atherosclerosis induced by a high-cholesterol and high-fat diet in the inbred strain of the Wuzhishan miniature pig. Anim Biotechnol.

[17] Dong X, Tsung HC, Mu YL, Liu LX, Chen HP, Zhang L, Wang HJ, Feng ST (2014). Generation of chimeric piglets by injection of embryonic germ cells from inbred Wuzhishan miniature pigs into blastocysts. Xenotransplantation.

[18] Khatri M, Richardson LA. Therapeutic potential of porcine bronchoalveolar fluid-derived mesenchymal stromal cells in a pig model of LPS-induced ALI. J Cell Physiol. 2018;233(7):5447-57. 10.1002/jcp.26397 PMC5878711 29231967

[19] Lu T, Hui X, Wang K, Wang S, Ma Y, Guan W (2014). Isolation and characterization of adipose-derived mesenchymal stem cells (ADSCs) from cattle. Appl Biochem Biotechnol.

[20] Bai L, Lennon DP, Caplan AI, Dechant A, Hecker J, Kranso J, Zaremba A, Miller RH (2012). Hepatocyte growth factor mediates MSCs stimulated functional recovery in animal models of MS. Nat Neurosci.

[21] Groth A, Ottinger S, Kleist C, Mohr E, Golriz M, Schultze D, Bruns H, Mehrabi A, Schemmer P, Büchler MW (2012). Evaluation of porcine mesenchymal stem cells for therapeutic use in human liver cancer. Int J Oncol.

[22] Ambrosi C, Manzo M, Baubec T (2017). Dynamics and context-dependent roles of DNA methylation. J Mol Biol.

[23] Tang Y, Liu L, Sheng M, Xiong K, Huang L, Gao Q, Wei J, Wu T, Yang S, Liu H (2015). Wip1 knockout inhibits the proliferation and enhances the migration of bone marrow mesenchymal stem cells. Exp Cell Res.

[24] Huang L, Niu C, Willard B, Zhao W, Liu L, He W, Wu T, Yang S, Feng S, Mu Y (2015). Proteomic analysis of porcine mesenchymal stem cells derived from bone marrow and umbilical cord: implication of the proteins involved in the higher migration capability of bone marrow mesenchymal stem cells. Stem Cell Res Ther.

[25] Yang Y, Liang G, Niu G, Zhang Y, Zhou R, Wang Y, Mu Y, Tang Z, Li K (2017). Comparative analysis of DNA methylome and transcriptome of skeletal muscle in lean-, obese-, and mini-type pigs. Sci Rep.

[26] Yang Y, Zhou R, Mu Y, Hou X, Tang Z, Li K (2016). Genome-wide analysis of DNA methylation in obese, lean, and miniature pig breeds. Sci Rep.

[27] Li R, Yu C, Li Y, Lam TW, Yiu SM, Kristiansen K, Wang J (2009). SOAP2: an improved ultrafast tool for short read alignment. Bioinformatics.

[28] Liu N, Williams AH, Maxeiner JM, Bezprozvannaya S, Shelton JM, Richardson JA, Basselduby R, Olson EN (2012). microRNA-206 promotes skeletal muscle regeneration and delays progression of Duchenne muscular dystrophy in mice. J Clin Investig.

[29] Zhang Y, Liu T, Meyer CA, Eeckhoute J, Johnson DS, Bernstein BE, Nussbaum C, Myers RM, Brown M, Li W (2008). Model-based analysis of ChIP-Seq (MACS). Genome Biol.

[30] Da WH, Sherman BT, Lempicki RA. Systematic and integrative analysis of large gene lists using DAVID bioinformatics resources. Nat Protoc. 2009;4(1):44-57. 10.1038/nprot.2008.211 19131956

[31] Mathias E, Nelson MR, Patrick S, Marc Z, Triantafillos L, George X, Cantor CR, Field JK, Dirk VDB (2005). Quantitative high-throughput analysis of DNA methylation patterns by base-specific cleavage and mass spectrometry. Proc Natl Acad Sci U S A.

[32] Smith ZD, Chan MM, Humm KC, Karnik R, Mekhoubad S, Regev A, Eggan K, Meissner A (2014). DNA methylation dynamics of the human preimplantation embryo. Nature.

[33] Jones PA (2012). Functions of DNA methylation: islands, start sites, gene bodies and beyond. Nat Rev Genet.

[34] Cottrell SE (2004). Molecular diagnostic applications of DNA methylation technology. Clin Biochem.

[35] Mark M, Rijli FM, Chambon P (1997). Homeobox genes in embryogenesis and pathogenesis. Pediatr Res.

[36] Erlandsen H, Ames JE, Tamkenath A, Mamaeva O, Stidham K, Wilson ME, Perez-Pinera P, Deuel TF, Macdougall M (2012). Pleiotrophin expression during odontogenesis. J Histochem Cytochem.

[37] Majore I (2011). Growth and differentiation properties of mesenchymal stromal cell populations derived from whole human umbilical cord. Stem Cell Rev Rep.

[38] Anveden Å, Sjöholm K, Jacobson P, Palsdottir V, Walley AJ, Froguel P, Al-Daghri N, Mcternan PG, Mejhert N, Arner P (2012). ITIH-5 expression in human adipose tissue is increased in obesity. Obesity.

[39] Murata Y, Konishi M, Itoh N (2011). FGF21 as an endocrine regulator in lipid metabolism: from molecular evolution to physiology and pathophysiology. J Nutr Metab.

[40] Alexei K, Wroblewski VJ, Anja K, Yun-Fei C, Clutinger CK, Tigno XT, Hansen BC, Shanafelt AB, Etgen GJ (2007). The metabolic state of diabetic monkeys is regulated by fibroblast growth factor-21. Endocrinology.

[41] Stejskal D, Ruzicka V (2008). Cardiotrophin-1. Rev Biomed Pap.

[42] Freed DH, Cunnington RH, Dangerfield AL, Sutton JS, Dixon IM (2005). Emerging evidence for the role of cardiotrophin-1 in cardiac repair in the infarcted heart. Cardiovasc Res.

[43] Daxing X, Crystal G, Jian Z, Rey-Chen P, Haifeng Z, Luyang Y, Vessella RL, Wang M, Jer-Tsong H (2009). DAB2IP coordinates both PI3K-Akt and ASK1 pathways for cell survival and apoptosis. Proc Natl Acad Sci U S A.

[44] Toyota M, Ho C, Ohe-Toyota M, Baylin SB, Issa JP (1999). Inactivation of CACNA1G, a T-type calcium channel gene, by aberrant methylation of its 5′ CpG island in human tumors. Cancer Res.

[45] Lin XJ, Shang XY, Manorek G, Howell SB. Regulation of the epithelial-mesenchymal transition by Claudin-3 and Claudin-4. PLoS One. 2013;8(6):e67496. 10.1371/journal.pone.0067496 PMC3689737 23805314

[46] Geng Z, Peijun H, Jochen G, B Michael G, Thomas R, Yfantis HG, Dong H, lee NH, H Richard A, S Perwez H (2013). FOXL1, a novel candidate tumor suppressor, inhibits tumor aggressiveness and predicts outcome in human pancreatic cancer. Cancer Res.

[47] Feng-Qiang Y, Feng-Ping Y, Wei L, Min L, Guang-Chun W, Jian-Ping C, Jian-Hua H, Jun-Hua Z (2014). Foxl1 inhibits tumor invasion and predicts outcome in human renal cancer. Int J Clin Exp Pathol.

[48] Ishii M, Asano K, Namkoong H, Tasaka S, Mizoguchi K, Asami T, Kamata H, Kimizuka Y, Fujiwara H, Funatsu Y (2012). CRTH2 is a critical regulator of neutrophil migration and resistance to polymicrobial sepsis. J Immunol.

[49] Giguere PM, Billard MJ, Laroche G, Buckley BK, Timoshchenko RG, McGinnis MW, Esserman D, Foreman O, Liu P, Siderovski DP (2013). G-protein signaling modulator-3, a gene linked to autoimmune diseases, regulates monocyte function and its deficiency protects from inflammatory arthritis. Mol Immunol.

[50] Kubin M, Cassiano L, Chalupny J, Chin W, Cosman D, Fanslow W, Mullberg J, Rousseau AM, Ulrich D, Armitage R (2001). ULBP1, 2, 3: novel MHC class I-related molecules that bind to human cytomegalovirus glycoprotein UL16, activate NK cells. Eur J Immunol.

